# A comparative study of cerebral anatomy teaching based on 3D body mobile application versus traditional teaching

**DOI:** 10.3389/fnana.2026.1883985

**Published:** 2026-09-11

**Authors:** Hao Wu, Wenwen Gao, Yingying Cheng, Haikang Zhao, Zhihai Yuan

**Affiliations:** Neurosurgery Department, The Second Affiliated Hospital of Xi'an Medical University, Xi'an, China

**Keywords:** 3D body mobile app, cerebral anatomy, comparative study, neurosurgery, traditional teaching

## Abstract

**Objective:**

To observe the application effect of the 3D Body Mobile Application (App) in the teaching of neuroanatomy.

**Methods:**

100 students from the outstanding class of clinical medicine major of Xi’an Medical University were selected as the research subjects and randomly divided into the experimental group (50 students) and the control group (50 students). During the teaching of cranial brain anatomy, the control group adopted the traditional teaching mode, while the experimental group combined the traditional teaching mode with the Three-dimensional (3D) Body Mobile App. After the two groups completed the teaching of cranial brain anatomy, a test paper was designed to analyze and statistically analyze the test scores. At the same time, the satisfaction of the teaching method’s interest, flexibility, richness of teaching content, and learning efficiency was investigated separately; finally, 20 peer clinical teachers conducted peer teaching evaluations from aspects such as teaching novelty, teaching logic, teaching rigor, teaching target orientation, student concentration, value orientation guidance, and integration of course ideological education.

**Results:**

In terms of the interestingness of teaching methods, the flexibility, richness, and learning efficiency of teaching content, the control group had the following percentages: 64, 54, 44, and 38%, respectively; the experimental group had the following percentages: 90, 84, 88, and 82%, respectively. The experimental group outperformed the control group (*p* < 0.05). Regarding teaching satisfaction and peer teaching evaluation, the experimental group scored higher than the control group, and the difference was statistically significant (*p* < 0.05). For average practical test scores and theoretical test scores, the experimental group scored higher than the control group, and the difference was statistically significant (*p* < 0.05).

**Conclusion:**

Using the 3D Body Mobile application in cranial anatomy teaching can enhance the interest and intuitiveness of instructional content to a certain extent. Through three-dimensional models, interactive operations, and dynamic demonstrations, previously abstract anatomical structures become vivid, intuitive, and easier to understand. This tool optimizes teaching methods and the allocation of educational resources, offering students a more flexible and self-directed learning approach. At the same time, it addresses some shortcomings of traditional teaching methods, providing new strategies and references for future instruction.

## 1 Introduction

In recent years, with the continuous development and deep integration of computer technology, virtual simulation technology, and three-dimensional imaging technology, a powerful and user-friendly three-dimensional anatomy learning software-the 3D Body Mobile App-has emerged (Chen et al., 2025). This software utilizes advanced image generation and three-dimensional modeling technologies to present various three-dimensional anatomical structures of the human body and has achieved high-precision three-dimensional reconstruction of the entire nervous system (Kumar et al., 2025). From the perspective of educational theory, its application aligns closely with the core principles of cognitive load theory. Through intuitive, visual three-dimensional models, it effectively reduces the extraneous cognitive load imposed by traditional two-dimensional anatomical images or textual descriptions, directing learners’ attention toward key structures and their spatial relationships. This frees up more cognitive resources for deep processing and understanding, thereby optimizing the management of intrinsic cognitive load (Sravanam et al., 2023). The neuroanatomical model it provides not only features strong interactive simulation capabilities but also offers rich structural detail, delivering a highly intuitive and visual learning platform for skull anatomy instruction (Sharma et al., 2025). This interactive model enhances the development of spatial cognitive ability: students can rotate, scale, and section the model from any angle, thereby mentally constructing a spatial mapping from two-dimensional representations to three-dimensional forms. As a result, their understanding and retention of complex anatomical spatial relationships improve significantly, and their mastery of anatomical knowledge is strengthened (Klitzman, 2022). Furthermore, the application of this software promotes the integration of active learning and multimedia learning. During use, students transition from passive recipients of knowledge to active constructors of personal understanding-exploring, virtually dissecting, and reorganizing anatomical models. This approach aligns with the constructivist principle of “learning by doing” (de Boer et al., 2022). Simultaneously, in accordance with the dual-coding principle of multimedia learning theory, the synchronous presentation of images, text, and audio activates both visual and auditory channels, thereby expanding working memory capacity and facilitating information integration and long-term retention (Sotgiu et al., 2020). Overall, this technological tool enhances students’ understanding and retention of complex anatomical spatial relationships, strengthens their mastery of anatomical knowledge, and improves the overall quality and effectiveness of anatomy instruction (Cole, 2025). Moreover, the portability of the 3D Body Mobile App affords students considerable autonomy in learning, enabling self-directed study anytime and anywhere outside class. This compensates for the temporal and spatial constraints of traditional classroom instruction and supports the planning and implementation of personalized learning pathways-further reinforcing learning outcomes (Smith et al., 2022).

In the era of artificial intelligence, we should adopt a “foundational knowledge first, technology-enabled enhancement” learning paradigm: preserving the rigor of systematic knowledge construction while fully leveraging the intuitive advantages of visualization technologies. Thus, rather than abandoning traditional learning methods, we should thoughtfully integrate emerging technologies. Judiciously applying artificial intelligence functionalities to support learning will be the critical final step (Moxham et al., 2015; Joshi et al., 2025). In practice, this means integrating the logical rigor of traditional anatomy textbooks with the dynamic interactivity of digital tools-using technology to lower entry barriers while maintaining systematic review of foundational concepts. Ultimately, through optimized cognitive load management, enhanced spatial cognition, promotion of active learning, enriched multimedia presentation, and empowerment of autonomous learning, the 3D Body Mobile App functions not merely as a teaching tool, but as a catalyst for reshaping the educational ecology of anatomy-providing robust support for cultivating medical professionals with profound spatial reasoning abilities and independent exploratory competencies (Guo et al., 2023).

## 2 Materials and methods

### 2.1 General information

The students from the outstanding class of clinical medicine major of Xi’an Medical University in the 2020 academic year were selected as the research subjects. They were randomly divided into the experimental group and the control group, with 50 students in each group. Experimental group: 26 males and 24 females; age range 17–20 years old, with an average age of (18.38 ± 4.11) years. Control group: 27 males and 23 females; age range 17–20 years old, with an average age of (18.21 ± 4.06) years. There was no statistically significant difference in general information such as gender and age between the two groups (*p* > 0.05), and they were comparable (The students included in the study were all familiar with the application of the software before the class began).

### 2.2 Methods

The same instructor conducted the inter-session practical teaching for the experimental group and the control group in neurosurgery. The teaching materials used were the 9th edition of “Surgery” (edited by Chen Xiaoping) and the 9th edition of “Systemic Anatomy” (edited by Bai Shulein). In the control group teaching, the traditional teaching mode was adopted, which mainly relied on textbook theoretical explanations, combined with PPT images, and appropriately used human organ model teaching aids. In the experimental group teaching, the traditional teaching mode was applied, and the 3D Body Mobile App was used in addition. The 3D Body Mobile App is divided into 5 modules: “Systemic Anatomy,” “Local Anatomy,” “Sectional Anatomy,” “Anatomy Micro-lessons,” and “Self-study.” It provides a full three-dimensional digital model of the human body, over 6,000 human structures, covering all human anatomical systems. After opening the software, click to select the human body part, view the corresponding anatomical structure, and then through the buttons “Display,” “Hide,” “Transparency,” and “Independent Display” in the menu area, you can achieve operations such as hiding, showing, transparency, and independent display, achieving a combination of reality and virtuality. For example, taking the brain as an example, this APP can detail display the scalp, bones, bone connections, muscles, nerves, lymph, circulation, etc. of the anatomical model, and can be combined with multi-point touch control to achieve random dragging, translation, display, separation, structure hiding, rotation and zooming, with high modeling accuracy and high realism, and can achieve top-down, bottom-up, etc. observation effects of each anatomical structure. At the same time, this software is combined with the teaching chapters, pre-sets the magnetic tiles of brain anatomy, classifies the local anatomy content, and systematically configures corresponding CT and magnetic resonance images based on the sectional specimen images, which can conveniently and quickly call up the digital human anatomy model, and provide a large amount of high-quality teaching resources through magnetic tiles, sectional images, micro-lesson videos, pictures, animations, etc.

1. The duration of the intervention: one semester.
2. The number of teaching sessions: 10 classes.
3. The amount of exposure time to the application: at least 1 hour every day.
4. App usage instructions: during class, the APP is used collectively. After class, students review independently (Each student is allowed to install apps on their own mobile phones.).
5. Outside class hours: unlimited.
6. Teaching condition: the two groups had the same teaching conditions and were equipped with multimedia. The same instructor conducted the teaching for both the control group and the experimental group separately.
7. The version information and developer details of the 3Dbody application are 8.9.30 and Third Person (Shanghai) Digital Technology Co., Ltd.

### 2.3 Observation indicators

#### 2.3.1 Examination scores

After the two groups of students completed the teaching of nervous system anatomy, both an experimental examination and a theoretical examination were administered. The full score for each examination was 100 points. After the students took the examinations, the scores for each examination were analyzed separately using statistical methods.

#### 2.3.2 Student teaching evaluation

Similarly, after the two groups of students completed the teaching of brain anatomy, a self-designed questionnaire was designed (The designed questionnaire has been verified and reviewed by supervisory experts, all of whom are senior experts in the field of surgery at Xi’an Medical University (a total of 20 experts, with an average age of 55 years and all holding the titles of chief physician and professor), and have over 10 years of teaching experience.). The two groups of students were taken as the survey subjects, and the satisfaction levels of the teaching method flexibility, the richness of teaching content, and the learning efficiency were investigated and analyzed. Each survey item was divided into three levels: satisfied, basically satisfied, and dissatisfied. The satisfaction rate was the sum of the satisfied rate and the basically satisfied rate.

#### 2.3.3 Peer teaching evaluation

Twenty peer clinical teachers conducted peer teaching evaluations. They investigated and analyzed the teaching novelty, teaching logic, teaching rigor, accuracy of key and difficult points grasping, students’ concentration, value orientation guidance, and the integration degree of course ideological education from these aspects.(Each item is scored out of 10 points).

The peer clinical teachers are senior experts in the field of surgery in Xi’an Medical University (The teaching experience exceeds 10 years, with an average age of 55. All the professionals hold the titles of chief physician and professor.).

### 2.4 Statistical methods

The data were analyzed using SPSS 20.0 statistical software. Quantitative data were expressed as (x̄ ± s), and *t*-tests were used for comparison; qualitative data were presented as rates, and *χ*^2^ tests were used for comparison. A *p* value less than 0.05 was considered statistically significant.

## 3 Results

### 3.1 Student teaching evaluation

In terms of the interestingness of teaching methods, the flexibility, richness, and learning efficiency of teaching content, the control group had the following percentages: 64, 54, 44, and 38%, respectively; the experimental group had the following percentages: 90, 84, 88, and 82%, respectively. The experimental group outperformed the control group, with statistically significant differences (*p* < 0.05) (Table 1).

**Table 1:** Compare the teaching effects of the two groups.

Group	Interestingness	Flexibility	Richness	Learning efficiency
Experimental group (*n* = 50)	45 (90%)	42 (84%)	44 (88%)	41 (82%)
Control group (*n* = 50)	32 (64%)	27 (54%)	22 (44%)	19 (38%)
*χ* ^2^	9.543	10.519	21.569	20.167
*p*	<0.01	<0.01	<0.01	<0.01

The teaching effect of the experimental group was significantly higher than that of the control group (*p* < 0.05) (Table 2).

**Table 2:** Compare the teaching satisfaction of the two groups.

Group	Satisfaction	General	Not satisfied	Degree of satisfaction
Experimental group (*n* = 50)	44	2	4	46 (92%)
Control group (*n* = 50)	31	5	14	36 (72%)
*χ* ^2^				6.775
*p*				<0.01

### 3.2 Peer teaching evaluation

In the peer evaluation of teaching, regarding the scores for teaching novelty, teaching logic, teaching rigor, teaching target orientation, student concentration, value orientation guidance, and the integration of course ideological and political content, the experimental group scored higher than the control group in the following three items: teaching novelty, logic, and student concentration, with *p* < 0.05 indicating statistically significant differences (Table 3).

**Table 3:** Compare peer teaching evaluation.

Evaluation content	Experimental group (*n* = 50)	Control group (*n* = 50)	*t*	*p*
Novelty	9.23 ± 0.43	6.24 ± 0.43	34.767	<0.001
Logicality	8.92 ± 0.55	6.07 ± 0.44	28.612	<0.001
Rigor	8.56 ± 0.67	5.07 ± 0.32	33.237	<0.001
Goal oriented	9.34 ± 0.38	4.07 ± 0.66	48.931	<0.001
Student concentration	9.36 ± 0.23	4.09 ± 0.74	48.088	<0.001
Value orientation	9.00 ± 0.43	5.07 ± 0.62	36.830	<0.001
IDCIPE	9.55 ± 0.58	9.43 ± 0.68	0.949	>0.05

Integration degree of course ideological and political education: IDCIPE.

### 3.3 Comparison of experimental examination scores before and after learning

The average score of pre-enrollment experimental examination scores in the experimental group was (72.50 ± 0.30) points, while that of the control group was (72.4 ± 0.20) points (*p* > 0.05). After the learning process, we found that the scores of the experimental group were (95.00 ± 0.55), which were higher than those of the control group (85.50 ± 0.75), and the differences were statistically significant (*p* < 0.05) (Table 4).

**Table 4:** Comparison of experimental examination scores before and after learning.

Scores	Experimental group (*n* = 50)	Control group (*n* = 50)	*t*	*p*
Pre-enrollment practical test scores	72.50 ± 0.30	72.4 ± 0.20	1.961	>0.05
Practical test after study	95.00 ± 0.55	85.50 ± 0.75	72.227	<0.001
*t*	−253.950	−119.338		
*p*	<0.001	<0.001		

### 3.4 Comparison of theoretical examination scores before and after learning

The average score of theoretical examination scores in the experimental group was (65.5 ± 0.20) points, while that of the control group was (65.6 ± 0.30) points (*p* > 0.05). After the learning process, we found that the scores of the experimental group were (92.2 ± 0.70), which were higher than those of the control group (83.60 ± 0.60), and the differences were statistically significant (*p* < 0.05) (Table 5). After systematic learning, the scores of the experimental group were better than those of the control group (*p* < 0.05) (Table 6).

**Table 5:** Comparison of theoretical examination scores before and after learning.

Scores	Experimental group (*n* = 50)	Control group (*n* = 50)	*t*	*p*
Pre-enrollment theoretical test scores	65.5 ± 0.20	65.6 ± 0.30	−1.961	>0.05
Theoretical test scores after study	92.2 ± 0.70	83.60 ± 0.60	65.959	<0.001
*t*	−259.333	−189.737		
*p*	<0.001	<0.001		

**Table 6:** Compare experimental examination and theoretical examination scores of the two groups.

Scores	Experimental group (*n* = 50)	Control group (*n* = 50)	*t*	*p*
Practical test scores	95.00 ± 0.55	85.50 ± 0.75	72.227	<0.001
Theoretical test scores	92.2 ± 0.7	83.60 ± 0.60	65.959	<0.001

## 4 Discussion

The nervous system is the core regulatory system for the body’s functions, playing an indispensable leading regulatory role in maintaining the internal environment stability, responding to external stimuli, and coordinating various life activities (Lewis et al., 2014). However, due to the complex anatomical structure of the nervous system and the numerous related professional concepts and academic terms, the biological mechanism of its functional regulation is abstract and obscure. Most medical students encounter varying degrees of learning and comprehension difficulties when studying the anatomy of the nervous system. Therefore, teachers teaching the anatomy of the nervous system need to continuously explore efficient and practical new teaching methods to improve the overall teaching quality of the nervous system anatomy. In recent years, with the rapid development of computer technology, three-dimensional reconstruction imaging technology has been widely applied in the field of biomedical engineering. 3D human anatomy mobile application software developed for anatomy teaching has been developed and gradually promoted and applied in the anatomy teaching fields at home and abroad (Nogueira et al., 2026). This software is a professional teaching tool based on virtual reality technology for human anatomy, covering multiple core areas of basic medicine such as anatomy and physiology, and providing digital virtual simulation teaching resources for sub-specialized directions such as traditional Chinese medicine meridians, movement function anatomy, step-by-step surgical simulation, and emergency operations (Poltronieri et al., 2026). This software not only contains comprehensive and accurate anatomical basic data sets but also has flexible interactive operation functions. Its functional completeness and technical advancement are at the leading level among similar products in China and abroad. Relying on the real-time three-dimensional interactive operation supported by this software, learners can observe the fine structures of various anatomical systems and major organs of the human body in a stereoscopic and intuitive manner (Fireman and de Assis Neto, 2025; Morris et al., 2026; Ramsay et al., 2026; Wang et al., 2025). The operation effect simulates the process of surgical knife layer-by-layer peeling the specimen in real anatomical operations, and can completely and clearly restore the entire process of human anatomy operations (Fang et al., 2026; Kanwal et al., 2024).

This study adopts a controlled research design to verify the teaching effect of this tool: in the teaching process of the anatomy of the nervous system, the control group adopts the traditional teaching mode for lectures, and the experimental group conducts auxiliary teaching based on the traditional teaching mode with this 3D human anatomy mobile application software. Statistical analysis results show that the average practical examination scores and average theoretical examination scores of the experimental group are better than those of the control group, and the difference is statistically significant (*p* < 0.05); in the satisfaction survey of the flexibility of teaching methods, the richness of teaching content, and learning efficiency, the satisfaction score of the experimental group is significantly higher than that of the control group, and the difference is statistically significant (*p* < 0.05); in the evaluation dimension of classroom concentration, the score of the experimental group is also higher than that of the control group, and the difference is statistically significant (*p* < 0.05). Although the learning effectiveness of the students in the experimental group in this study has been clearly improved, the generation of this result is partly related to the novelty of the teaching plan, the mobilization of learners’ subjective initiative, the improvement of classroom attention level, and the Hawthorne effect, etc. In summary, introducing 3D human anatomy mobile application software as a digital auxiliary teaching tool in the teaching practice of the anatomy of the nervous system can to some extent improve the intuitiveness and three-dimensional presentation effect of teaching content. This teaching mode not only compensates for the shortcomings of traditional teaching methods, converts abstract and complex neural anatomical structures into vivid and clear, easy-to-understand visual content, but also optimizes the allocation and utilization of teaching resources, and can provide learners with a self-accessible autonomous learning platform. The above improvements can effectively promote students’ understanding and mastery of the anatomy of the nervous system in the short term, improve students’ learning efficiency, and have achieved positive and significant effects in improving students’ academic performance and overall teaching satisfaction (Berkmen et al., 2025; Hindy et al., 2026; Patera et al., 2025; Mapes, 2025; Krungkraipetch et al., 2026). However, the long-term application effectiveness of this method still needs further in-depth research to verify. Furthermore, a systematic review and meta-analysis indicated that virtual reality technologies can be used as supplementary teaching tools in anatomy education to enhance educational quality and optimize the learning process. The study also showed that augmented reality (AR) technologies can serve as supportive tools in anatomy instruction, although their teaching effectiveness was not significantly superior to traditional teaching methods (Salimi et al., 2024).

## 5 The limitations of the research

### 5.1 Sample limitations

This study’s sample was solely derived from a single medical college, which may not represent the overall situation of medical students with different educational backgrounds and learning abilities across the country or globally. The sample size was relatively limited, and factors such as grade, gender, and learning style were not considered to affect the research results, restricting the generalizability of the research conclusions.

### 5.2 Technical application limitations

3D body movement applications (such as 3D anatomical models, augmented reality technology) vary in hardware equipment, operating system compatibility, screen resolution, and touch response speed, which may affect students’ learning experience and application results. Additionally, some students may have inaccurate assessment of learning effectiveness due to unfamiliarity with technology or low reliance on mobile devices.

### 5.3 Short-term nature of teaching intervention

This study only compared short-term teaching effects and did not set up a long-term tracking observation mechanism, making it impossible to assess the long-term effects of 3D mobile applications on knowledge consolidation and the transformation of clinical practice abilities. Key indicators such as memory retention curves and knowledge transfer abilities were not included in the research scope.

### 5.4 Limitations of assessment tools

The knowledge structure assessment tools used may be subjective. For example, self-assessment questionnaires and knowledge test questions may not fully cover the core knowledge points of brain anatomy, and they did not stratify assessment of different levels of cognitive abilities (such as memory, understanding, application, analysis, and evaluation). It is difficult to comprehensively reflect the differences in teaching effects.

### 5.5 Insufficient control of confounding variables

The study did not adequately control confounding variables such as students’ extracurricular self-study time, prior knowledge of brain anatomy, learning motivation, and differences in teaching styles of teachers. These factors may significantly affect the final research results and lead to biased estimations of the effectiveness of teaching interventions.

### 5.6 Differences in teaching environment and resources

During the experiment, the traditional teaching group and the 3D mobile application teaching group may be affected by uneven distribution of teaching resources (such as model quantity, teacher guidance time, and differences in laboratory equipment) which may affect the fairness of the research results. Additionally, factors such as learning atmosphere and frequency of teacher-student interaction in different teaching environments were not effectively controlled.

### 5.7 Insufficient assessment of interactivity and immersion

The user satisfaction assessment of 3D mobile applications in terms of interaction design, immersion, and visual feedback is relatively brief, lacking systematic analysis of user experience. For example, whether students’ learning effectiveness is affected by complex operation interfaces, graphic rendering delays, or lack of stereoscopic perception, this has not been fully explored.

### 5.8 Uniqueness of teaching effect measurement

The study mainly focused on changes in knowledge structure (such as knowledge completeness, accuracy, and relevance), but did not pay attention to changes in non-cognitive factors such as students’ interest in brain anatomy learning, learning attitude, and learning confidence. The comprehensive assessment dimensions of teaching effects need to be expanded.

### 5.9 Limitations of control group design

The traditional teaching group may adopt different teaching methods (such as lecturing, discussion, model demonstration, etc.), and the standardization of teaching content and activities is difficult to ensure, resulting in differences in teaching interventions between the two groups other than technical applications, which may also include other teaching factors, thereby affecting the reliability of the research conclusions.

## 6 Conclusion

The 3D Body Mobile Application can enhance the quality of craniocerebral anatomy education in the short term, optimize the learning process, and provide learners with a more flexible and independent learning approach. However, the long-term teaching effectiveness of this application requires further in-depth research for validation. At present, it is recommended to use it as a supplementary teaching tool in craniocerebral anatomy teaching.

## Data Availability

The original contributions presented in the study are included in the article/supplementary material, further inquiries can be directed to the corresponding author.
